# Terahertz reflectivity dataset: Reading text on both sides of the page

**DOI:** 10.1016/j.dib.2026.112978

**Published:** 2026-06-19

**Authors:** Geetika Vadali, Hugo Cherid, Erwan Émile, Haolian Shi, Leor Jacobi, D.S. Citrin, Cédric Pradalier, Alexandre Locquet

**Affiliations:** a College of Computing, Georgia Institute of Technology, Atlanta, Georgia 30332 USA; b School of Electrical and Computer Engineering, Georgia Institute of Technology, Atlanta, Georgia 30332-0250 USA; c Georgia Tech-CNRS IRL 2958, Georgia Tech-Europe, 2 Rue Marconi, 57070 Metz, France; d Université de Lorraine, Ile du Saulcy, 57000 Metz, France; e Department of Jewish Art, Faculty of Jewish Studies, Bar-Ilan University, Ramat-Gan 5290002 Israel

**Keywords:** Terahertz time-of-flight tomography, Nondestructive evaluation, Signal processing, Machine learning, Ink classification

## Abstract

Non-destructively reading text on a sheet of paper with characters on both sides, when those characters might be obscured, unreadable visually, and the paper cannot be turned over, remains an open problem in document analysis. This problem has practical relevance to archival characterisation, fragmentology, and forgery detection. Terahertz (THz) time-of-flight tomography (TOFT) offers the ability to penetrate paper and parchment as well as sensitivity to the composition of inks. However, progress in data-driven THz analysis has been hindered by the absence of publicly available, labelled reference datasets. While a range of historical materials have been employed in manuscripts and printed books, it is favorable to phrase the problem in a way that is accessible to numerous laboratories to develop techniques to extract such texts. In view of this aim, we introduce a curated THz-TOFT dataset comprising raster-scan measurements of four distinct print/writing media (henceforth ink), namely toner, inkjet, iron gall ink, and graphite, deposited on standard copy paper. Samples were prepared from contemporary materials consisting of one sheet of standard copy paper with overlapping test patterns or characters on each side. THz-TOFT data were acquired in reflection mode under controlled, nitrogen-purged conditions, yielding per-pixel time-domain waveforms. Multiple checkerboard-style test patterns are provided per ink type, each covering four ink configuration classes: no ink, ink on the front, back, and on both surfaces. Raw data is distributed as CSV files encoding spatial coordinates, full time-domain waveforms (reflected THz electric field), and manually validated class labels. To demonstrate the suitability of the dataset for machine-learning applications, a convolutional neural network baseline is provided together with a PyTorch-compatible dataloader. This baseline is trained and tested separately for each of the ink datasets, and the predictions on unknown data are qualitatively evaluated to compare the characteristic media properties.

Specifications Table

**Table:** 

Subject	Engineering; Applied Machine Learning
Specific subject area	Terahertz time-of-flight tomography and imaging of paper and ink for nondestructive evaluation and machine-learning benchmarking.
Type of data	Tabular Data containing labelled, spatially cropped A-scans of specific patterns in paper-ink combinations.
Data collection	A commercial time-domain THz system (TeraPulse Lx, TeraView Ltd., UK) was used in reflection mode to acquire raster-scanned 2D data from paper samples bearing four ink types (iron gall, graphite, laser-printer toner, inkjet). Scans cover ∼50×50 mm2 with 0.5 mm step size; each pixel stores a 2048-point time-domain THz electric field with a sampling duration of 0.02 ps. All measurements were performed in a nitrogen-purged enclosure.
Data source location	Data collected at Georgia Tech-CNRS IRL 2958, Georgia Tech-Europe, 2 Rue Marconi, 57070 Metz, France.
Data accessibility	Repository name: Terahertz Scans of Patterned Paper Sheets with Multiple Ink Types Data identification number (DOI): https://doi.org/10.57745/AWN8Y5 Direct URL: https://entrepot.recherche.data.gouv.fr/dataset.xhtml?persistentId=doi:10.57745/AWN8Y5
Related research article	None

## 1 Value of the Data

• These data constitute, to the best of the authors’ knowledge, the first publicly available, spatially resolved, labelled THz-TOFT dataset covering four distinct ink types (iron gall, graphite, laser toner, and inkjet) deposited on a standard paper type and acquired under nominally identical, standardised conditions. This fills a recognised gap in the field where existing studies are application-specific and lack intercomparability.
• The dataset provides a controlled benchmark resource that enables objective comparison of ink classification and characterisation methods across research groups, removing the need for every team to collect their own data from scratch. Within this controlled setting, the samples provide inter- and intra-material variation without claiming full representativeness of archival or forensic documents.
• The labelled, structured format with per-pixel 2048-point time-domain waveforms and a four-class labelling scheme (no_ink, ink_front, ink_back, ink_both), is directly compatible with supervised machine-learning pipelines, supporting the growing application of deep learning to THz-TOFT data.
• The four ink types span a broad range of material properties (surface-fused versus fibre-penetrating deposition, carbon-based versus iron-bearing chemistry, electrically insulating versus semiconductive character), making the dataset relevant to diverse research communities including cultural heritage science, archival document analysis, and modern document forensics.

## 2 Background

THz TOFT has emerged as a powerful noninvasive and nondestructive tool for the analysis of documents, art, and archaeological objects, offering sensitivity to material composition through the penetration depth, low photon energy, micron-scale resolution, and broad spectral bandwidth characteristic of the THz frequency range [1]. The optical properties of paper in this regime have been extensively characterised; THz attenuation and refractive index spectra have been shown to depend on cellulose fibre orientation, density, and water content [2]. Additionally, spectral changes caused by natural and artificial aging provide quantitative markers of document degradation [3]. Beyond the paper support, THz imaging has demonstrated sensitivity to a range of writing and printing media: carbon-based inks including lamp black and graphite produce strong image contrast [4]; iron gall inks exhibit reproducible absorption bands at THz frequencies [5]; and graphite shows Drude-like free-carrier absorption which is sensitive to its structure [6]. Furthermore, THz imaging can be performed on stacks of multiple paper sheets due to the transparency of many paper types in the THz band [7]. These properties have been exploited in the study of manuscripts, documents, and books with applications ranging from the decipherment of hidden manuscript text [8] to the separating content across stacked paper layers [9] and super-resolution imaging of document surfaces [10]. THz imaging holds promise to advance the field of fragmentology [11], allowing for the nondestructive reading of text on reused manuscript and book fragments in book bindings. THz imaging employs nonionising radiation and is expected to be scalable for scanning large quantities of materials.

Despite this progress, the field faces a critical infrastructural gap: lack of a publicly available, standardised THz dataset for paper and ink characterisation. Existing studies characterise inks in isolation or under application-specific conditions, with limited ink diversity in any single work. Gibson et al. [12] demonstrated the complementarity of THz with other modalities for document imaging but found that no single technique robustly detects all ink types, highlighting the need for well-characterised, per-modality reference data. Meanwhile, the growing application of machine learning to THz imaging [[13], [14], [15], [16], [17], [18], [19]] demands labelled and structured datasets for training and evaluation, yet researchers currently must collect these from scratch, which creates a substantial barrier to entry.

## 3 Data Description

### 3.1 Dataset overview

The dataset comprises 2D raster-scan THz-TOFT measurements of four inks deposited on standard 80 g/m2 copy paper ∼100μm thick. The inks or marking methods are black laser toner, black inkjet, iron gall ink, and graphite. Each scan is stored as a CSV file in which every row corresponds to one A-scan: the first two columns encode the (*x, y*) spatial coordinates (mm), the next 2048 columns contain the time-domain THz electric-field values, and a final column carries the four-class ink label that represents the spatial location of deposited ink with respect to the paper.

**Fig. 1:**
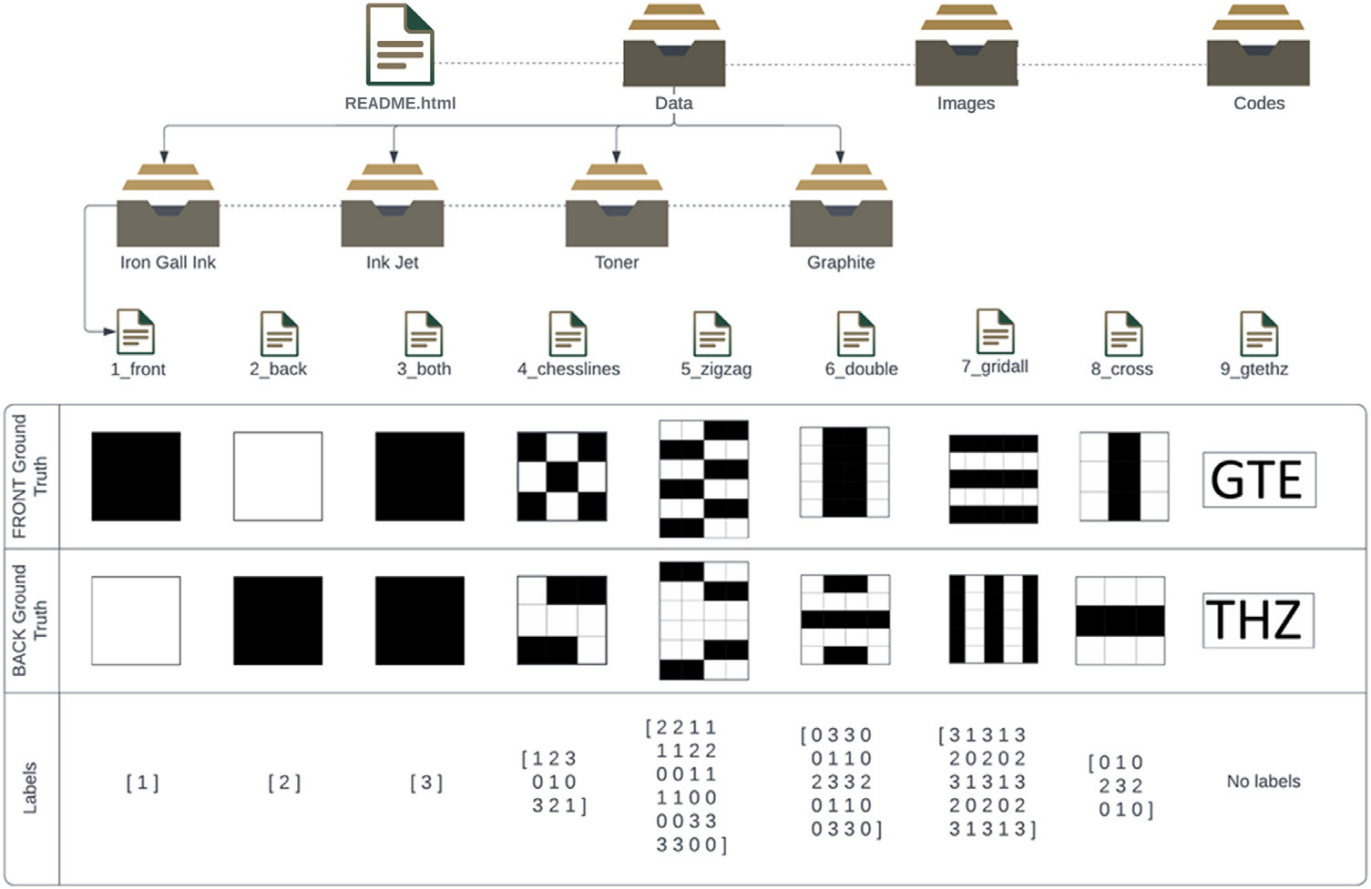
Organization of data along with individual patterns and mapped labels for each file.

For consistent labelling throughout the process, from initial labelling to downstream classification and inference, the following mapping is used: {0: no_ink, 1: ink_front, 2: ink_back, 3: ink_both}. Fig. 1 illustrates the data organisation and labelling patterns.

The dataset consists of four ZIP files, each containing individual pattern CSVs per ink type. A README.html file summarizes the data and its metadata, including information about the materials, their sources, terahertz machine specifications, a concise description of the dataset structure, instructions for extracting files, and directions to the code scripts.

In Codes.zip, we provide scripts that cover the full pipeline of our baseline machine learning model, including results and instructions for running and reproducing them. For data processing, the dataloader script takes .csv files as input and extracts windowed spatial patches ready for training. Scripts for model architecture, training, and testing are included to facilitate reproducibility. Additionally, some visualisation scripts, already integrated into the test scripts, are available. These scripts can visualise C-scans, A-scans, and label scatter plots for robust and verifiable evaluation. Usage instructions for visualisations, training, validation, and testing are also detailed in the README.md. To further facilitate reproduction and transparency, we include all obtained results in a separate folder for each ink type. This includes the trained model, C-scan images (label-wise and grayscale), and validation confusion matrices across the four ink classes to evaluate performance and generalisability.

## 4 Experimental Design, Materials and Methods

### 4.1 Time-domain terahertz system

**Fig. 2:**
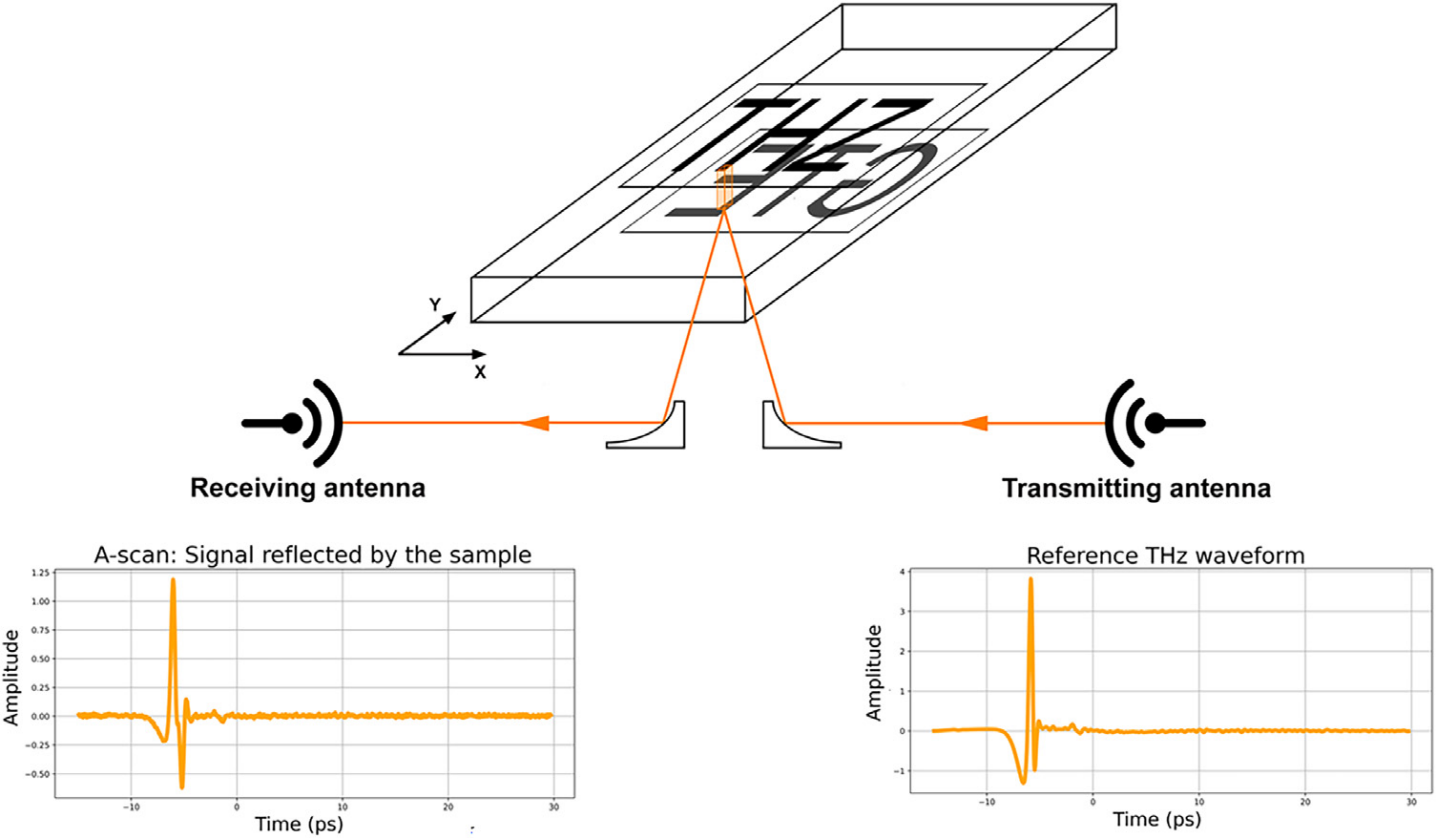
Schematic representation of the TeraPulse LX scanner from TeraView used in reflection mode.

Signals are acquired using a commercial time-domain THz system (TeraPulse Lx, TeraView Ltd., Cambridge, UK) in reflection mode. Utilising a near-infrared ultrafast fibre laser, the system generates THz pulses at an 80 MHz repetition rate with a bandwidth ranging from ∼0.1 to 6 THz. Two photoconductive antennas, triggered by ultrafast near-infrared pulses, perform emission and detection of the signal. The resulting THz pulses are quasi-single-cycle and last for a few *ps*. Low-numerical THz focusing optics direct the pulse onto the surface and acquire the reflected beam at near-normal incidence (depth of focus ∼ 5 mm, far exceeding sample thickness). More information about the THz system is given in Table 1, and the schematic representation of the Terapulse LX scanner is shown in Fig. 2.

**Table 1:** Terahertz scanning system parameters.

Parameter	Value
Sampling interval	0.02 ps
Laser pulse duration	≲ 100 fs
Laser repetition rate	80 MHz
Laser wavelength	780 nm
Optical conditions	f/1 optics
Operating humidity	20% - 70% Relative Humidity
Accessories	Reflection Imaging Module (RIM)

A raster scan is performed on the sample surface using the RIM extension from Teraview. At each point on the sample, the detected THz electric field is acquired as a function of time delay (A-scan). This A-scan consists of 2048 samples, spanning a temporal window of 0.02 ps per time sample. Most scans cover an area of ∼50×50 mm^2^ with a spatial step size of 0.5 mm in both lateral directions. Transverse resolution is limited by the low-numerical-aperture THz optics and varies across the high-signal-to-noise bandwidth. All time-domain waveforms are subsequently transformed into the frequency domain, yielding the complex spectral amplitude at each spatial coordinate. To mitigate strong absorption of THz radiation by atmospheric water vapor, all measurements were conducted in a nitrogen-purged enclosure. A metal coupon (an excellent THz broadband reflector) reference scan was acquired under identical conditions immediately before each sample scan. This enabled differential analysis and accounted for residual system drift. The resulting data volume is a 3D cube with two spatial dimensions and one time-delay dimension. C-scans are 2D maps formed (in the present context) at a constant time delay; while B-scans are cross-sections at constant *x* or *y* values.

## 5 Materials

### 5.1 Paper support

The support used throughout this study was Mondi Dolphin Everyday paper with a grammage of 80 g/m^2^ and a nominal thickness of 100 μm. This commercially available paper was kept constant across all samples and closely resembles many copy-paper brands widely available on the market.

### 5.2 Ink media

Four inks were selected to span a broad range of material compositions, deposition mechanisms, and application domains.

**Iron Gall ink**, a black ink widely used for over 2000 years, was included in this dataset partly due to the intense blacks it can achieve. The ink used has a unit specific gravity of 17. It comprises ferrous sulphate (FeSO_4_), tannic acid derived from oak galls, and gum arabic as a binder, consistent with historical manuscript practice [20]. Upon application and drying, iron(II) ions oxidise to iron(III) and form stable iron-tannate coordination complexes that chemically bond to cellulose hydroxyl groups, integrating with the fibre matrix. Iron gall ink was employed extensively by scribes who created manuscripts and early modern books [5,8]. In this study, the ink was applied to paper using a brush to create patterned samples.

**Graphite** (standard hard black pencil) consists of crystalline carbon in a layered hexagonal lattice, mixed with clay binder, and deposited by mechanical abrasion. This produces a thin, discontinuous layer of carbon flakes adhering to the uppermost paper fibres. The semimetallic electrical conductivity of graphite is expected to produce Drude-like absorption, which increases toward lower THz frequencies [6].

**Toner** (model Bizhub C3320i, manufactured by Konica Minolta) consists of fine thermoplastic polymer particles, predominantly a styrene-acrylate copolymer incorporating carbon black as the primary colorant, together with charge-control agents and flow additives. During printing, particles are electrostatically transferred and permanently fused by a heated roller, forming a thin, continuous polymer film on the paper surface.

**Inkjet** (model OfficeJet Pro 6230, manufactured by HP) is an aqueous carbon-black pigment-based ink deposited by thermal drop-on-demand jetting. Upon deposition the ink is absorbed into the paper fibre network, resulting in a spatially diffuse distribution of colorant within the substrate.

### 5.3 Data acquisition and labeling protocol

Each paper sample was affixed to a custom 100×100 mm^2^ 3D-printed scaffold prior to scanning. The scaffold holds the paper taut in a planar configuration, ensuring a consistent sample-to-detector distance throughout the raster scan. Samples were secured with tape at the edges, with the inked region centered within the scan window.

**Fig. 3:**
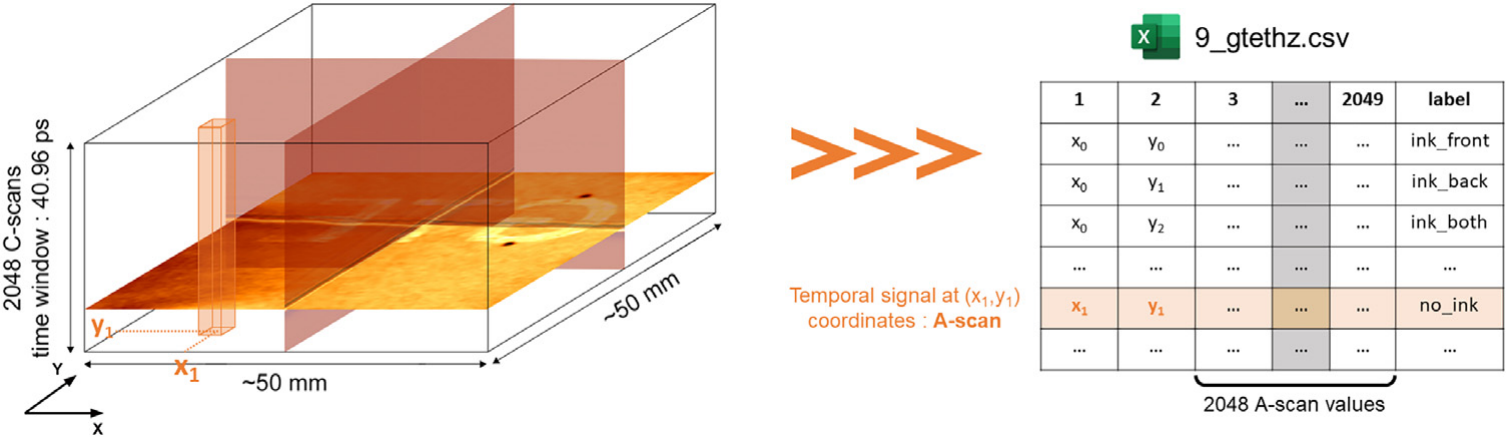
Representation of the data cube acquired with two spatial dimensions (*x,y*) and one temporal dimension (left). Example of the CSV file extracted from the acquired data cube (right).

Raw measurement data is exported from the THz system as binary .tprj project files. A custom extraction pipeline parses each file, recovering the per-coordinate amplitude-versus-time waveform for every (*x, y*) position. The four extreme corners of each sample are pin-pricked to manually specify the corner coordinates and, thus, the spatial extent of each scan. Extracted waveforms were saved as CSV files. Each row corresponds to one A-scan, with the first two columns encoding the *x* and *y* coordinates, followed by 2048 amplitude values and the label of this A-scan in the last column. This is illustrated graphically in Fig. 3.

A structured grid-based labelling scheme was applied to each scan. The scan area was divided into a user-specified number of rows and columns, and each grid cell was assigned one of four labels: no_ink, ink_front, ink_back, or ink_both. Since the patterns used for training (and that require labelling) are checkerboard-style geometric patterns, the grid-based labeling simplifies the process by assigning labels per row-column pair. A padding factor of approximately 0.125 is applied around the margins of all grid cells, where the labelling is less reliable than for interior coordinates. For character-containing scans where unambiguous labelling was not possible, no label was assigned, as in the *GTE-THZ* sample that we use solely for final qualitative testing. Labels are stored as an additional column in the CSV file.

### 5.4 Machine learning benchmark

To demonstrate the utility of the dataset and provide a reproducible baseline for future work, we present a set of benchmark classification experiments using the acquired THz-TOFT data. These experiments establish a reference performance against which future methods can be compared and illustrate the dataset’s potential in distinguishing ink/marking-media presence and surface location from the waveform data.

The benchmark is formulated as a four-class supervised classification problem. Given one waveform or a spatial patch of THz time-domain waveform(s) acquired at a pixel location on the sample surface, the task is to predict the ink label at that location from the set {no_ink, ink_front, ink_back, or ink_both}, corresponding to class indices {0,1,2,3} respectively. This formulation captures both the questions of the material presence (is ink present?) and its geometry with respect to the paper (on which surface of the paper does the ink reside?).

### 5.5 Data loading and preprocessing

As part of data loading, we perform signal window selection that isolates a 192-sample segment centered on the first reflection peak (96 samples on each side), reducing each raw 2048-sample waveform to the physically relevant surface-contact event. To exploit spatial correlations inherent in terahertz raster scans, signals are grouped into *P* × *P* spatial patches on a regularly discretised grid, recovered from the irregularly sampled measurements via nearest-neighbour projection. Peak alignment is performed on the patch mean signal, yielding labelled tensors of shape (*P*^2^, *L*) = (9, 192) per sample. During training, light augmentation via random time-shifts and amplitude scaling is applied to improve generalisation. A PyTorch-compatible dataloader is provided alongside the dataset to facilitate this.

### 5.6 Convolutional neural network model

The baseline model utilised is a compact three-block 1-D convolutional neural network (CNN) that operates directly on multi-channel terahertz time-domain signals. The model accepts a patch tensor of shape (*P*^2^ × 192), where P is the size of the signal-patch considered per data point, and produces a four-class output. Patterns *cross* and *chesslines* form the validation set, *GTE-THZ* is used for the final qualitative testing, and all the remaining patterns are used in the training process. Due to some variability in number of samples obtained per pattern per ink, this split ensures an average percentage spilt of 63:17:20 across the train, validation and test sets, respectively.

Each input sample consists of a 3×3 spatial patch, comprising 9 adjacent measurement points. Each point is represented as a time series of 192 samples, resulting in an input tensor of shape (9, 192). The network applies three successive 1-D convolutional layers with 7 filters and progressively smaller kernels (32, 16, and 8 samples respectively). Each layer is followed by ReLU activation and 2× max-pooling, reducing the temporal dimension by a factor of 8 before flattening. The resulting feature vector is passed through three fully connected layers (64 → 32 → *n*_classes_ = 4). The first two of these layers use ReLU activations, producing class logits over four ink-coverage categories. Training is conducted using the Adam optimizer (learning rate= 1 × 10^−5^) with cross-entropy loss over a maximum of 20 epochs. Early stopping is triggered after 10 consecutive epochs without improvement in validation accuracy. Scripts for data loading, training, testing, and visualising results are available in the dataset repository.

### 5.7 Per-Ink multi-class classification results

#### 5.7.1 Training and inference

As previously discussed, we conduct training and inference of the same model architecture separately for the four ink types. This approach helps the machine-learning models discern subtle signal differences due to the presence or absence of ink and its spatial location relative to the paper.

The training set consists of files such as front, back, both, zigzag, double and gridall. Validation set consists of the files cross and chesslines. We test the trained models on GTE-THZ sample to qualitatively visualise the model’s prediction on double-sided overlapping characters.

We conduct downsampling-based class balancing on the training and validation sets to ensure an equal number of data points per class and reduce bias caused by imbalance. Table 2 presents the qualitative results of predictions for the four inks on the same pattern: a single sheet of paper with GTE on the front side and THZ on the back side, overlapping. Since the text is written left-to-right and the front (GTE) faces the scanner, the text on the front appears inverted relative to the back. The table shows predictions for the paper’s front side (combining ink-front and ink-both) and for the back side (combining ink-back and ink-both). This representation is grayscale, based on the softmax of predictions, and is post-processed by merging classes (front and both for front side, back and both for back side) to more closely approximate the ground truth image.

**Table 2:** Prediction results of trained multi-class classification models on unseen GTE-THZ pattern data, per ink-type.

Ink Type	Front Detection (GTE)	Back Detection (THZ)	Best Final Validation Accuracy (%)
Iron Gall Ink			56
Graphite			90
Inkjet			30
Toner			39

Quantitatively, Table 2 also presents the best final validation accuracies obtained by training our models for 20 epochs, consistent with the previously mentioned settings. Both the quantitative and qualitative results are mutually consistent in their ability to visualise the predicted pattern. Graphite achieves the best predictive and classification performance over our current baseline, followed by iron gall ink, toner and inkjet. Inkjet completely fails, predicting only slightly better than random classification. Iron gall ink struggles with areas that have no ink or ink on the opposite side, explaining the noise in negative regions. Toner prediction indicates that the model finds it difficult to discern information from the front and back sides of the paper, explaining the overlapping predictions.

## Limitations

The current dataset is limited in scale, representing only four inks on a single paper type, with a modest number of scan patterns per ink type. Therefore, it is intended as a controlled benchmarking resource rather than a fully generalisable representation of archival or forensic documents. Nonetheless, the selected materials are standard and widely available, providing a useful basis for modelling more complex cases. While the dataset is motivated by applications to historical documents, all samples were prepared from contemporary, commercially available materials; the extent to which findings generalise to aged paper, historical inks, and degraded substrates remains to be established. Expanding the dataset to include additional paper grades, ink formulations, historical paper, aged samples, and a wider variety of printing technologies and acquisition environments would further increase its generalisability. The spatial step size of 0.5 mm somewhat limits transverse resolution, which may affect the precision of ink boundary localisation. While moderately better transverse resolution might be achieved using high-numerical-aperture THz optics, this would come at the cost of a lower depth of focus. Furthermore, the manual corner-coordinate specification during the extraction pipeline introduces a potential source of positional uncertainty and highlights an opportunity for more automated data processing pipelines.

## Ethics Statement

Our study does not involve human subjects, animal experiments, or data collected from social media platforms. The authors confirm that the current work adheres to the ethical requirements for publication in Data in Brief as detailed in the journal’s Guide for Authors.

## Credit Author Statement

**G.V.:** Data Curation, Formal Analysis, Investigation, Methodology, Validation, Visualisation, Writing - Original Draft; **H.C.:** Data Curation, Investigation, Validation, Writing - Review & Editing; **E.E.:** Investigation, Validation, Writing – Review & Editing; **H.S.:** Data Curation, Conceptualisation, Validation; **L.J.:** Conceptualisation, Investigation, Materials, Writing – Review & Editing; **D.S.C.:** Conceptualisation, Writing - Review & Editing; **C.P.:** Methodology, Supervision, Writing - Review & Editing. **A.L.:** Methodology, Resources, Supervision, Writing - Review & Editing, Funding Acquisition.

## Data Availability

Recherche Data GouvTerahertz Scans of Patterned Paper Sheets with Multiple Ink Types (Original data).
